# Evaluation of home detection algorithms on mobile phone data using individual-level ground truth

**DOI:** 10.1140/epjds/s13688-021-00284-9

**Published:** 2021-06-02

**Authors:** Luca Pappalardo, Leo Ferres, Manuel Sacasa, Ciro Cattuto, Loreto Bravo

**Affiliations:** 1 Institute of Information Science and Technologies (ISTI), National Research Council (CNR), Pisa, Italy; 2 Faculty of Engineering, Universidad del Desarrollo, Santiago, Chile; 3 Telefónica R&D, Santiago, Chile; 4 University of Turin, Turin, Italy; 5 ISI Foundation, Turin, Italy

**Keywords:** Mobile phone data, Data science, Human mobility, Home location detection

## Abstract

Inferring mobile phone users’ home location, i.e., assigning a location in space to a user based on data generated by the mobile phone network, is a central task in leveraging mobile phone data to study social and urban phenomena. Despite its widespread use, home detection relies on assumptions that are difficult to check without ground truth, i.e., where the individual who owns the device resides. In this paper, we present a dataset that comprises the mobile phone activity of sixty-five participants for whom the geographical coordinates of their residence location are known. The mobile phone activity refers to Call Detail Records (CDRs), eXtended Detail Records (XDRs), and Control Plane Records (CPRs), which vary in their temporal granularity and differ in the data generation mechanism. We provide an unprecedented evaluation of the accuracy of home detection algorithms and quantify the amount of data needed for each stream to carry out successful home detection for each stream. Our work is useful for researchers and practitioners to minimize data requests and maximize the accuracy of the home antenna location.

**Supplementary Information:**

The online version contains supplementary material available at 10.1140/epjds/s13688-021-00284-9.

## Introduction

Nowadays, there is a strong demand by all branches of government, including national statistical offices, to invest in projects that explore how we can integrate novel digital data into all kinds of official statistics (e.g., mobility, immigration, epidemic control). This is brought about by the need to reduce costs and increase timeliness of data collection, potentially providing faster data-driven decisions to sophisticated societal problems [20, 41, 42]. In particular, in recent years, we have witnessed the emergence of methodologies that use heterogeneous digital data sources - such as social media data, GPS traces or mobile phone records - to estimate socio-economic indicators of objective and subjective well-being [11, 14, 16, 22, 25–27, 31, 36, 39, 45].

The many applications of “big data” analytics to any kind of official statistics depend critically on our ability to identify, with more or less error, *where someone lives*, i.e., detecting an individual’s home location. This impacts all aspects of the work on statistics with non-traditional data sources such as the estimation of population density [10, 18, 38], commuting and migration flows [5, 15, 17, 19, 28], air pollution [21, 37], and the estimation of privacy risk [8, 9, 12, 32, 33], and is of special importance now to inform epidemic models of COVID-19 transmission [34]. The knowledge of the home location of individuals forms the crucial link between digital data and census data, making it a key enabler for the integration of these two sources of information.

Most of the home detection algorithms (HDAs) proposed in the literature [1, 13, 43, 44] process mobile phone records according to *ad-hoc* heuristics rather than principled approaches. Indeed, they rely on simple decision rules based on how much, and when, an individual calls in each location during the period of observation. The simplest category of HDAs identifies an individual’s home location as the one in which they made the highest number of calls. A variant of this algorithm identifies an individual’s home location as the one in which they make the highest number of calls during nighttime (e.g., between 7 pm and 7 am). Other HDAs use a combination of criteria or slight variations of the ones mentioned above [43, 44]. Although these algorithms have been used in many works and tools [6, 7, 11, 24, 29–31, 35, 46], a thorough validation of their accuracy is still missing.

One reason for this is that, with few exceptions [1, 13], ground truth data at the individual level are not provided by mobile providers for privacy protection reasons, making it difficult to obtain a large enough sample of users for which complete information about positions and residence are available at the same time. For example, Vanhoof et al. [43, 44] provide a high-level validation of the most popular HDAs by comparing each mobile phone tower’s population as estimated by official censuses with the number of users whose home is detected to be in that tower. They conclude that there is an urgent need for validation of HDAs at the individual level, i.e., evaluating the performance in detecting the home location on a set of individuals for which the actual home location is known [43, 44].

It’s important to notice, also, that HDAs have been validated mostly on Call Detail Records (CDRs) [4, 23], which describe each user’s position only when they make or receive a call. Since the inter-event time between two calls is bursty [40], CDRs are sparse and provide an incomplete picture of an individual’s positions over time [2], and it has become more so in recent years, since calls have decreased noticeably, and communication apps (e.g., Whatsapp, Zoom, Telegram, Discord) have increased. It is not clear, however, whether eXtended Detail Records (XDRs), which are generated partly by the individual and partly by the phone device (thus indirectly accounting for the above mentioned apps), or Control Plane Records (CPRs), which are purely triggered by the mobile phone network, overcome the limitations of using the more temporally sparse CDRs, providing more accurate estimates of an individual’s home location.

This paper provides an attempt at a fine-grained validation of HDAs on individual-level ground truth data and three streams of mobile phone records – CDRs, XDRs, and CPRs. Specifically, 65 users working for Telefónica Chile gave their written consent to provide us access to their phone records for two weeks, as well as their actual address of residence. This information allowed us to correctly assess the accuracy of HDAs, i.e., their capacity to detect a user’s actual home correctly, on a ground truth dataset. Our validation reveals the most accurate HDA among the popular ones proposed in the literature, and that XDRs and CPRs improve the accuracy of HDAs considerably with respect to CDRs. Moreover, we set up a data minimization experiment to study how the accuracy of detecting home locations changes by the stream used and the number of records for each user. We find that, depending on the stream, just a small fraction of the records is enough to achieve reasonably accurate estimations of an individual’s home location, hence providing a tool to manage the uncertainty and utility trade-off in geo-privacy.

We believe our individual-level validation is a useful and timely contribution to the field, which paves the road towards the definition of more accurate home detection algorithms, and a much needed standardized method for home detection that could make studies more comparable.

## Mobile phone datasets

We obtained written consent from 65 people working for Telefónica Chile to use the precise latitude and longitude of their homes in Santiago de Chile, calculated over the original addresses (e.g., 123 Santiago Street, Providencia) with reverse lookup using Google Maps. We also obtained consent to gather two weeks (14 days, September 24-October 6, 2019 inclusive) of their historical mobile phone records for three streams: Call Detail Records (CDRs), eXtended Detail Records (XDRs), and Control Plane Records (CPRs). For privacy purposes, no other demographic information was either requested nor volunteered. It is worth mentioning that this is not a dataset of participants in a controlled study (we did not give them phones, or SIM cards, for example). Instead, it is a rather uncontrolled sample of Telefónica employees (among thousands of employees), who consented to have the two weeks of their telco records accessed for research purposes, and linked to their residence address. As consent was obtained for accessing past records, the mobile phone traces comprising our dataset are, at least in this dimension, more “ecologically valid”, as opposed to the more common experimental setting in which participants know they will be monitored over a future time interval.

**Figure 1:**
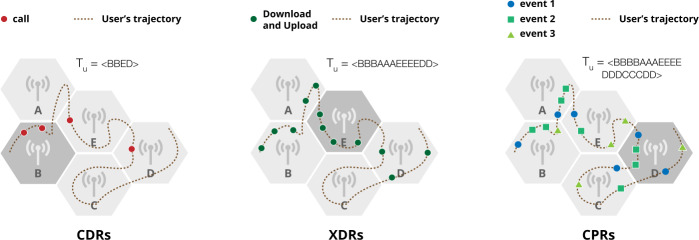
Illustrative example of Call Detail Records (CDRs), eXtended Detail Records (XDRs) and Control Plane Records (CPRs) of a user *u*. The hexagons represent mobile phone towers and black dots the positions where the user starts a call (CDRs), a download/upload operation (XDRs), or a network event (CPRs). Red dots indicate calls, green dots indicate download/upload operations, blue dots, green squares and green triangles indicate network events. The dotted line indicates the real movement of the user, from the left to the right, within each panel. The dark grey tower indicates the tower in which the user has the highest number of records. From this example, we observe that: 1) CDRs are the most sparse and CPRs the most dense data stream; 2) when *u* does not perform any call or download/upload operation in the area covered by a tower, there is no information about the user’s position in CDRs and XDRs; 3) CPRs is the only type of records that can describe all the towers *u* passed through

Mobile phone operators collect CDRs and XDRs for billing and operational purposes. CDRs are purely user-triggered, i.e., they are generated by the users every time they make or receive a phone call. Consequently, when a user does not make/receive a call, their position is not recorded. XDRs are a mixture of human- and device-triggered, either by explicitly requesting an http address or automatically downloading content from the Internet (e.g., emails). In contrast, CPRs are network-triggered (e.g., assigning a new antenna, connecting new devices) and are used to monitor the cellphone network status. Although all three streams are equal in terms of their geo-location properties (the set of towers remains more or less constant in time), they vary significantly in their time granularity and data sparsity. Figure 1 shows an illustrative example of CDRs, XDRs, and CPRs of an individual, and how the most frequented tower changes stream by stream. At the Telco level, there was no aggregation of any time-event associated to any of the streams. The only pre-processing step was the pseudo-anonymization of the phone numbers (i.e., the phone numbers were hashed).

**Figure 2:**
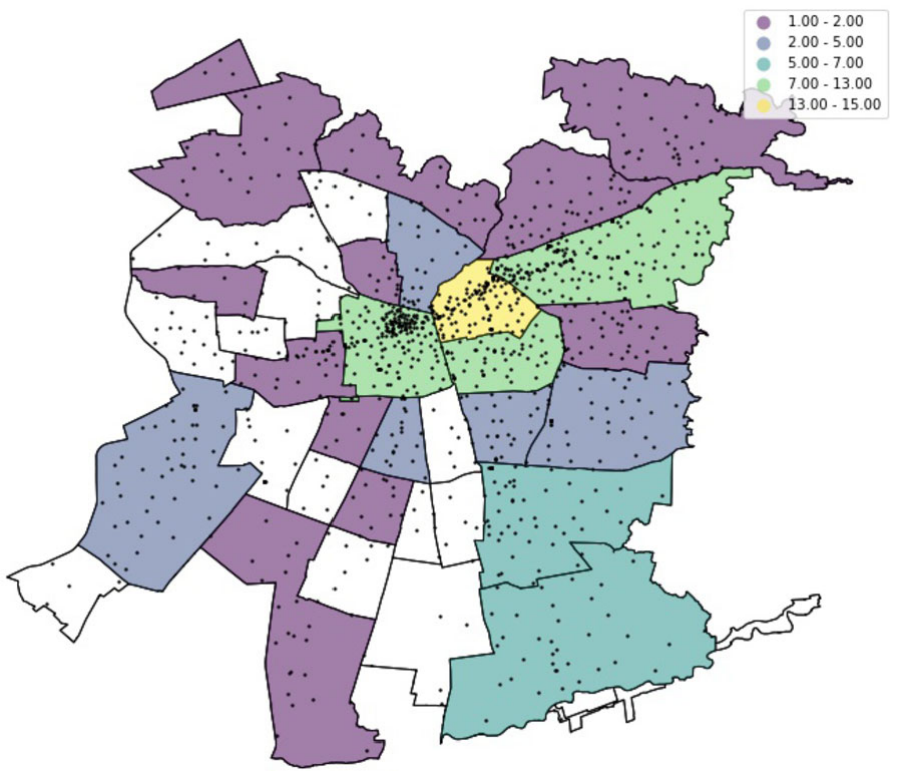
Position of antennas (black dots) and presence of our 65 users in the largest urban area of Santiago de Chile. Antennas cluster in regions with more demand, and become sparser in regions with less demand. The noticeable line of antennas in the middle of the map are those that fall in the wealthier/financial comunas (a large administrative unit, represented in our map with black lines)

Formally, a CDR is a tuple $(n_{o},n_{i},t,d,A_{o},A_{i})$, where $n_{o}$ and $n_{i}$ are the identifier of the user that places the call (the caller) and the one that receives the call (the callee), respectively; *t* is a timestamp of when the call was placed and *d* is the call’s duration (in minutes). $A_{o}$ and $A_{i}$ are the antennas for the outgoing call (where the call is placed) and the receiving call (where the call is picked up), respectively. Our dataset contains 1663 antennas (Fig. 2), which may be a tower (containing many antennas), or a single antenna, as for example in indoor spaces [3]. An antenna is a tuple $A=(l_{x}, l_{y})$ where $l_{x}$, and $l_{y}$ are the longitude and latitude of *A*. For simplicity, from now on we always use the term “tower” to denote a location.

An XDR is a tuple $(n,t,A,k)$; in contrast to CDRs, there is only one antenna *A* involved, *n* is the caller’s identifier, *t* is a timestamp of when the record is created and *k* is the amount of downloaded information (in kilobytes). Finally, a CPR is a tuple $(n,t,A, e)$, where each $e \in E$ is an “event” of the network. There are many possible control plane events, such as “handovers” (when a new antenna is pushed on the device), the “(re-)activation” of a phone, etc.

**Figure 3:**
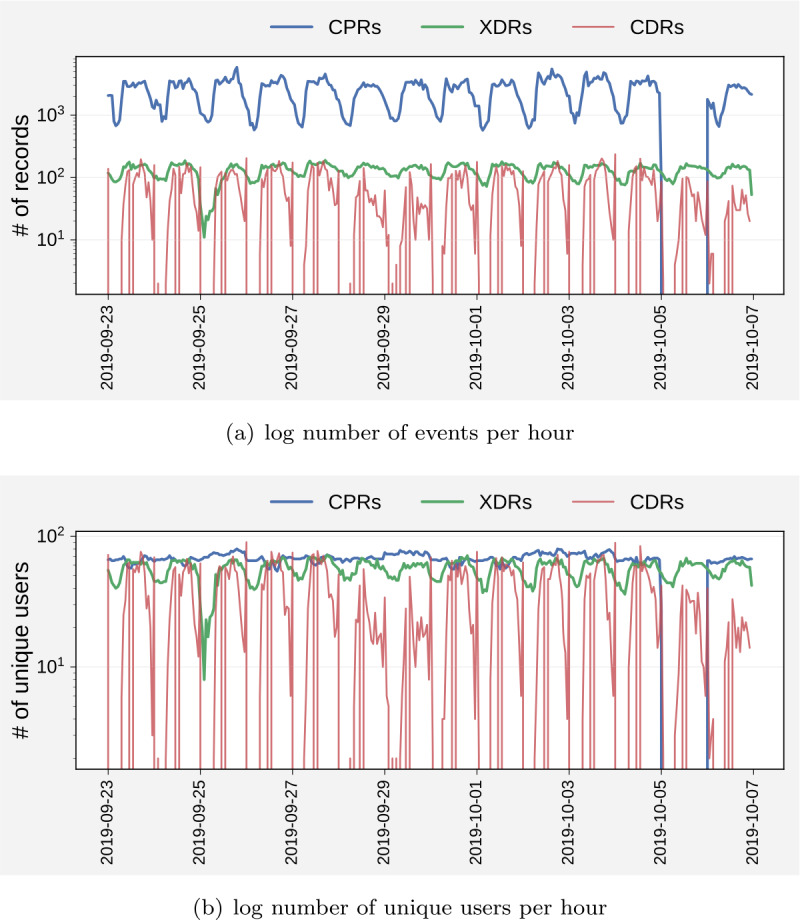
A comparison of the number of (**a**) records and (**b**) unique users for CDRs, XDRs and CPRs, for 14 days (except Oct 5 for CPRs, for which there was no data ingestion)

For the period under investigation, we have 19,234 CDRs, 43,607 XDRs and 772,871 CPRs. Figure 3a compares the number of records in our dataset of the three streams. For CPRs, October 5 is missing (see Fig. 3), and there are 13 days only. CPRs are the most frequent ones (i.e., most records per user), followed by XDRs and CDRs. CDRs are also the data type with the highest variance across the days of the week. Figure 3b shows, per each stream, the number of active users (those with at least one record) per hour. Again, CPRs show the highest number of active users, followed by XDRs and CDRs. Note also that CPRs have the lowest variance between the week’s days, while CDRs have the highest variance.

From the CDRs, XDRs, and CPRs, we compute the activity of each user in each tower according to 37 home detection algorithms (HDAs, see Sect. 3). Tables 1 and 2 show the structure of the aggregated data.

**Table 1:** Structure of the activity dataset. The records are sorted by device, stream, HDA, and activity

device	tower	activity	stream	HDA
afa64	0052	5	CDRs	MA
afa64	0056	3	CDRs	MA
afa64	0012	1	CDRs	MA
afa64	0052	2	CDRs	DD
afa64	0056	1	CDRs	DD
…	…	…	…	…

**Table 2:** Structure of the ground truth dataset. Each record describes a device and the three closest towers to the device owner’s actual home location

device	closest	2nd closest	3rd closest
afa64	0003	0087	0043
214ab	0022	0043	0011
1c75db	0065	0022	0021
f1599	0087	0076	0055
d666d	0003	0009	0087
…	…	…	…

In the Activity dataset, each row describes the activity a user has in a tower according to a data stream and an HDA. Column “device” indicates the (anonymized) device identifier. Column “tower” indicates the (hashed) identifier of a mobile phone tower. Column “activity” indicates the amount of activity the user has in the tower. Column “stream” indicates the type of records (CDRs, XDRs, or CPRs) and column “HDA” the home detection algorithm that calculated the activity. Given a stream *T*, a user *u*, and an HDA *X*, the row with the highest “activity” value is the home detected by *X* on *T* for *u*. For example, in Table 1, for user afa64, tower 0052 is the most active one according to HDA MA (Most Amount) using CDRs.

For XDRs and DD, the same column indicates the number of distinct days the user has at least one XDR for that tower. Table 1 shows some examples of records corresponding to a single user. In total, the Activity dataset contains 555,771 rows.

In the Ground truth dataset, each row describes a mobile phone device and the identifiers of the three closest towers to the user’s actual home location. Column “device” indicates the (anonymized) device identifier. Columns “closest”, “2nd closest”, and “3rd closest” indicate the identifiers of the towers that are, respectively, the closest tower, the second closest tower and the third closest tower to the user’s actual home location. These towers are identified using a *k*-nearest neighbor algorithm, with $k=3$. Table 2 shows some examples of ground truth records. In total, we have 65 rows in the Ground truth dataset.

## Results

We use the Activity dataset to detect the 65 users’ home locations according to a set of HDAs and use the Ground truth dataset to assess the HDAs’ accuracy. Specifically, we extend the set of HDAs defined for mobile phone data by Vanhoof et al. [43, 44]. For every stream - CDRs, XDRs, and CPRs - and for every user *u*, we calculate the most active towers given the specific criteria of each HDA.

Algorithm MA (“Most Amount”, we follow Vanhoof’s [43, 44] naming conventions here) counts the number of records at every tower for *u*. The tower with the highest activity is *u*’s home location. We consider two variants of this algorithm, which count the number of records at every tower during weekdays (MA-WK) and weekend days (MA-WE). Moreover, we consider an extension of MA that implements a spatial perimeter of 1 km around each tower (MA-R). The activity of a tower *x* is hence the sum of the activities of all towers within a radius of 1 km, including *x*.

Algorithm DD (Distinct Days) calculates the activity of towers for every *unique* day. Thus, wherever *u* is on most distinct days is identified as their home.

The algorithms based on the Time Constraints criterion (TC) calculates the most active towers during a specific time period. For example, Algorithm TC-19-9 counts the number of records at every tower between 7 pm and 9 am, inclusive. The tower that is most active during this period at night is identified as *u*’s home location. Clearly, many possible time periods may be specified during nighttime or daytime, which would lead to different variants of the TC algorithm. In our study, we consider eight time periods, four in nighttime (19-7, 19-9, 21-7, 21-9) and four in daytime (9-21, 9-19, 7-19, 7-21). Since the choice of the time period does not affect the results significantly, and for the sake of clarity, we present results for one period in nighttime (19-7) and on period in daytime (7-19) only, leaving the presentation of the results for all time periods in the Supplementary Material 1. It is important to notice that TC-WK-7-19 acts as a kind of “counterfactual”, since it is hard for people who work during regular business hours to be home during those times. Effectively, this is what happens, with TC-WK-7-19 consistently ranking as one of the worst HDAs (as we will show in Sect. 3.2), showing that HDAs are sensitive in these dimensions. This of course introduces a limitation of the current study; namely, the identification of home locations for people who work during nighttime. We discuss this briefly in the conclusions.

For each time period, we consider two further variants of TC, which consider records during weekdays (from Monday to Friday, TC-WK) and weekend days (Saturday and Sunday, TC-WE). This choice is motivated by the fact that call patterns are expected to change over the weekends, when users are not at work during daytime. Finally, for each time and week period, we consider an extension of TC that implements a spatial perimeter of 1 km around each tower, similarly to MA-R. Table 3 lists the HDAs we consider in our study.

**Table 3:** Characterization of the considered HDAs according to their criterion, week period, day period, and space radius

name	criterion	week period	day period	radius	description (home is tower where)
MA	Maximum Amount	all week	all day	-	most activities occurred
MA-WK	Maximum Amount	weekdays	all day	-	most activities occurred during weekdays
MA-WE	Maximum Amount	weekend days	all day	-	most activities occurred during weekend days
MA-R	Maximum Amount	all week	all day	1 km	most activities occurred in that tower and all towers within 1 km radius to it
DD	Distinct Days	all week	all day	-	maximum active days were observed
TC-19-7	Time Constraints	all week	nighttime	-	most activities occurred between 7 pm and 7 am (nighttime)
TC-7-19	Time Constraints	all week	daytime	-	most activities occurred between 7 am and 7 pm (daytime)
TC-WK-19-7	Time constraints	weekdays	nighttime	-	most activities occurred between 7 pm and 7 am (nighttime) and during weekdays
TC-WE-19-7	Time constraints	weekend days	nighttime	-	most activities occurred between 7 pm and 7 am (nighttime) and during weekend days
TC-WK-7-19	Time constraints	weekdays	daytime	-	most activities occurred between 7 am and 7 pm (daytime) and during weekdays
TC-WE-7-19	Time constraints	weekend days	daytime	-	most activities occurred between 7 am and 7 pm (daytime) and during weekend days
TC-R-19-7	Time constraints	all week	nighttime	1 km	most activities occurred between 7 pm and 7 am (nighttime) in all towers within 1 km
TC-R-7-19	Time constraints	all week	daytime	1 km	most activities occurred between 7 am and 7 pm (daytime) in all towers within 1 km

In the case of ties between the towers with the highest activity according to a certain HDA, we select the tower with the minimum collective activity, i.e., the sum of the activity of all users in that tower. If the towers have the same collective activity, we select a tower at random. We find that HDAs implementing a spatial perimeter of 1 km (e.g., MA-R, TC-R-19-7) generate the highest number of ties, and that CPRs and XDRs are more resilient to ties than CDRs (see Supplementary Information 1).

### Agreement between HDAs

We first investigate whether, for the same user, different HDAs detect the same home location, and how the choice of the stream influences the agreement between algorithms. Given a stream $T \in \{\mathrm{CDRs}, \mathrm{XDRs}, \mathrm{CPRs}\}$, we assess to which degree two HDAs *X* and *Y* agree on a set of individuals *G* by evaluating the Simple Matching Coefficient (SMC) [44], defined as:

1 $$ \mathrm{SMC}_{T}(X, Y) = 100 * \frac{\sum_{i=u}^{|G|} \delta (H_{X, T}(u), H_{Y, T}(u))}{N}, $$

where $u=1 \dots |G|$ denotes the 65 users in our dataset, $H_{X, T}(u)$ and $H_{Y, T}(u)$ denote the home location detected for user *u* on stream *T* by HDAs *X* and *Y* respectively, and *δ* is the Kronecker delta which is equal to 1 when $H_{X, T}(u) = H_{Y, T}(u)$. The Kronecker delta is 0 otherwise. Values of SMC thus range between 0 and 100 and can be interpreted as the percentage of individual cases for which both algorithms detected the same home location, i.e., the agreement between two HDAs.

**Figure 4:**
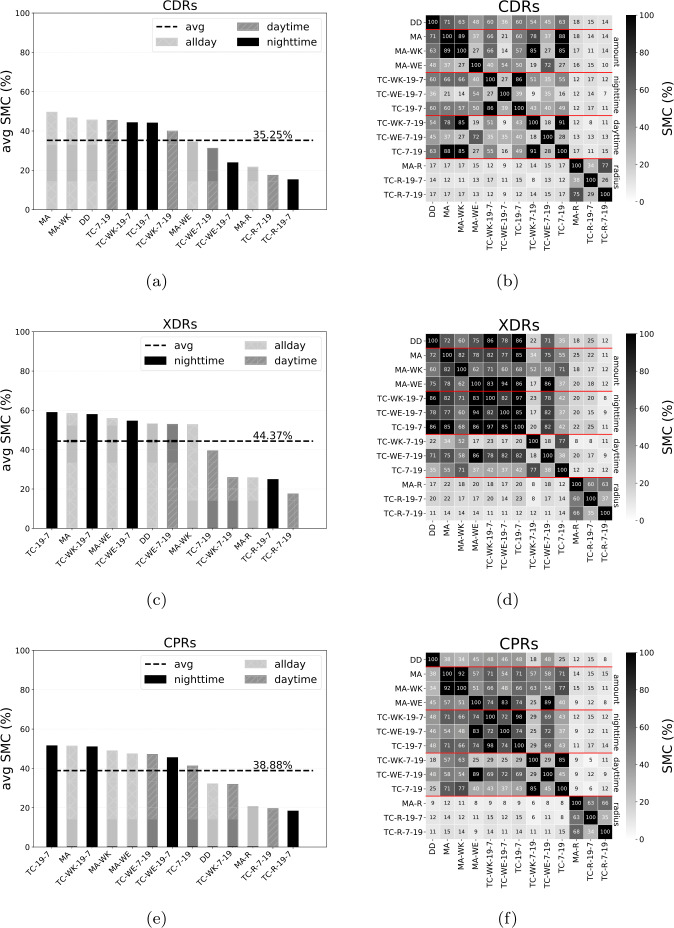
(**a**), (**b**), (**e**) The SMC of each pair of HDAs, for each stream. The black dashed line indicates the average SMC of a stream. (**b**), (**d**), (**f**) SMC between each pair of HDAs (elements of the matrix), for each stream. Each element of the matrix indicates the SMC between the two HDAs

Figure 4 shows the average SMC of the home detection algorithms (HDAs). Specifically, in Fig. 4a, c, e, we compare the HDAs that consider the number of records during nighttime, daytime, or all day. We find that the average agreement between the HDAs is highest for XDRs (44.37%), and lowest for CDRs (35.25%). Moreover, for XDRs and CPRs, TC-19-7, based on nighttime records, is the HDA with the highest average SMC, while it is MA for CDRs. Figure 4b, d, f shows the SMC between each pair of HDAs. HDAs based on radius (MA-R, TC-R-19-7, and TC-R-7-19) constitutes a separate group and achieve the lowest average SMC. Moreover, the HDAs based on nighttime records and on all-day records tend to have a higher average SMC than those based on daytime records. This agrees with the intuition that users in our sample will be working during day time, and at home during nighttime.

These results provide important practical insights. First, XDRs should be preferred when performing home location detection: since they lead to the highest agreement between HDAs, the use of XDRs would guarantee a higher reproducibility of the results. Unfortunately, most of the works in the literature use CDRs, and with different HDAs. Second, for XDRs, the average agreement is about 44%. This means that in more than half of the cases the HDAs disagree on what the home location of a user should be, highlighting that the choice of the HDA is crucial. The best solution would be to select the combination of HDA and stream that, on average, gives the most accurate home location detection. Therefore, what is the most accurate combination of HDA and stream on individual ground truth data?

### Accuracy of HDAs

For each user $u \in G$, where *G* is the set of the 65 users, we know the address of residence, from which we obtain the exact position (e.g., latitude and longitude) of their actual home location $H^{(u)}$ using Google Maps. Note that the closest tower is not necessarily the one that serves a user at home. Indeed, in some areas of the city where the density of towers is high (e.g., close to downtown), some antennas are turned off by the operator at different times of the day, or they become so heavily used that the network re-routs some users. Moreover, a user may have a tower close to their home, but with the azimuth of the antenna pointing to the opposite direction, while a tower farther away may be directly “illuminating” the user’s home. For these reasons, for each $u \in G$, we compute the three closest mobile phone towers, $H_{1}^{(u)}$, $H_{2}^{(u)}$ and $H_{3}^{(u)}$, to the ground truth position $H^{(u)}$.

**Figure 5:**
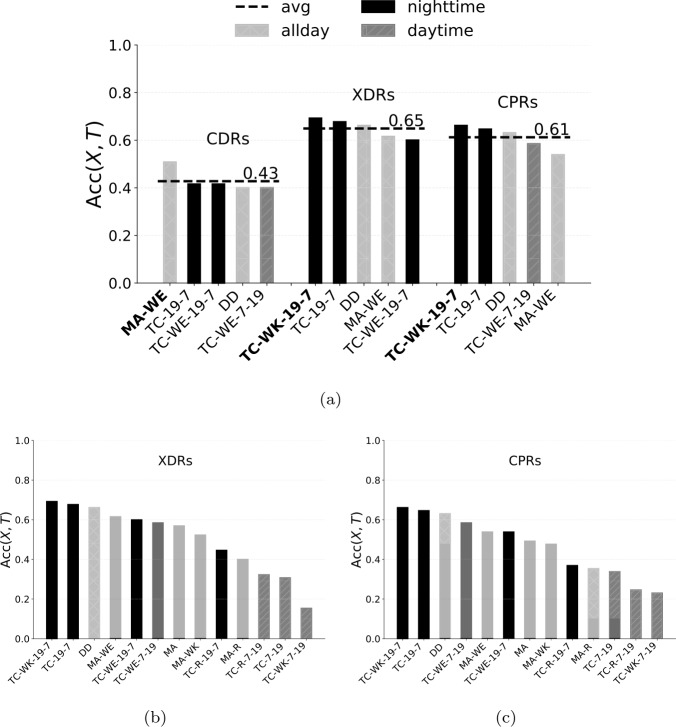
(**a**) Home detection accuracy of the top five HDAs for each stream. Dark bars indicate algorithms based on nighttime records, dark grey bars indicate those based on daytime records, light grey bars indicate those using records during all day. The dashed line indicates the average accuracy across the top five HDAs. In the x axis, we highlight in bold the best combination of stream and HDA. (**b**) Home detection accuracy of 13 HDAs for XDRs. (**c**) Home detection accuracy of 13 HDAs for CPRs

Given an algorithm *X*, a stream *T*, and a user *u*, we say that $H_{X, T}(u)$ is correct if $H_{X, T}(u) \in \{H_{1}^{(u)}, H_{2}^{(u)}, H_{3}^{(u)}\}$, i.e., if the home location detected by *X* on *T* is at least one of the three closest towers to *u*’s ground truth home location. Therefore, we define the *home detection accuracy* $\mathrm{ACC} _{X, T}$ of the combination of HDA *X* and stream *T* as the number of correctly classified home locations over the total number of ground truth users $|G|$. As an instance, $\mathrm{ACC} _{X, T} = 0.50$ means that, using stream *T*, algorithm *X* can correctly detect the home location for half of ground truth users. Table 4 shows the accuracy of each combination of stream and HDA, and Fig. 5a shows the accuracy of the top five HDAs for each stream (see Supplementary Information 2 for the accuracy of all HDAs).

**Table 4:** Accuracy of each combination of HDA and stream

	three nearest towers			nearest tower		
	CDRs	**XDRs**	CPRs	CDRs	XDRs	CPRs
DD	0.40	**0.66**	0.63	0.22	0.40	0.35
MA	0.26	**0.57**	0.49	0.15	0.34	0.25
MA-R	0.31	**0.38**	0.35	0.15	0.20	0.09
MA-WE	0.51	**0.62**	0.54	0.25	0.35	0.28
MA-WK	0.22	**0.52**	0.48	0.12	0.31	0.25
TC-19-7	0.42	**0.68**	0.65	0.25	0.43	0.40
TC-7-19	0.22	0.31	**0.34**	0.12	0.18	0.17
TC-R-19-7	0.31	**0.43**	0.38	0.11	0.22	0.11
TC-R-7-19	0.29	**0.32**	0.28	0.14	0.15	0.12
TC-WE-19-7	0.42	**0.60**	0.54	0.20	0.34	0.32
TC-WE-7-19	0.40	**0.58**	0.58	0.25	0.34	0.31
**TC-WK-19-7**	0.37	**0.69**	0.66	0.23	0.45	0.42
TC-WK-7-19	0.17	0.15	**0.23**	0.09	0.09	0.11
avg	0.33	**0.50**	0.47	0.18	0.29	0.24

From a stream perspective, XDRs lead to the highest average accuracy, $\overline{\mathrm{ACC}}_{X, \mathrm{ XDRs}} = 0.65$, slightly better than CPRs ($\overline{\mathrm{ACC}}_{X, \mathrm{ CPRs}} = 0.61$), and significantly better than CDRs ($\overline{\mathrm{ACC}}_{X, \mathrm{ CDRs}} = 0.43$). This is because, on the one hand, streams with finer temporal granularity (XDRs and CPRs) guarantee a larger number of observations than CDRs, also leading to fewer ties (see Supplementary Information 1). On the other hand, XDRs are favored by their mixed nature, since they are generated by either the user’s usage of the phone (e.g., explicitly a message through an app) or the mobile network connection (e.g., automatically downloading emails).

**Figure 6:**
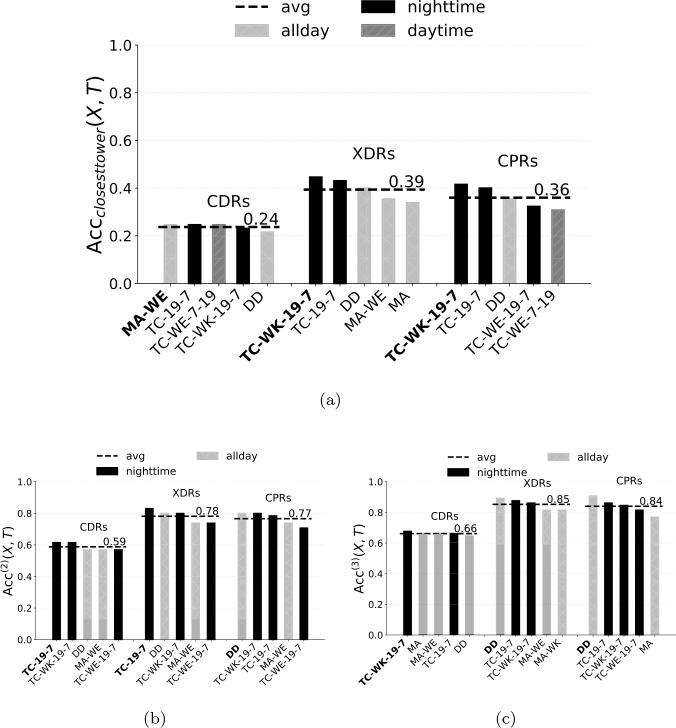
(**a**) Home detection accuracy of the top five HDAs considering just the nearest tower to the user’s actual home as ground truth. Dark bars indicate algorithms based on nighttime records, dark grey bars indicate those based on daytime records, light grey bars indicate those using records during all day. The dashed line indicates the average accuracy across the top five HDAs. In the x axis, we highlight in bold the best combination of stream and HDA. (**b**) Home detection 2-accuracy and (**c**) 3-accuracy, for the top five HDAs for each stream

Focusing on XDRs and CPRs, TC algorithms are the best ones, especially those based on weekdays and nighttime records (black bars in Fig. 5). In particular, TC-WK-19-7 is the HDA achieving the best accuracy overall ($\mathrm{ACC} _{ \mathrm{ TC-WK-19-7, XDRs}} = 0.69$), i.e., it detects the correct home location for almost 70% of the ground truth users. On the contrary, algorithms that implement the spatial perimeter (e.g., MA-R) and those based on daytime records (e.g., TC-7-19, see dark grey bars in Fig. 5) achieve the worst accuracy. In particular, TC-WK-9-19 is the worst HDA overall with $\mathrm{ACC} _{ \mathrm{ TC-WK-9-19, XDRs}} =0.11$, i.e., it detects the correct home location for just 10% of the users. Thus, there is a large variability of the accuracy among the considered combinations of streams and HDAs, highlighting how crucial their selection is. It is worth noting that the different nature of the streams reveals different behaviours of the ground truth users. While XDRs and CPRs do not necessarily require explicit actions by the users (hence, automatically capturing their position at home during nighttime), CDRs do require a deliberate user action (calls), revealing that users are more likely to make or receive calls during weekends (MA-WE) than during workdays at night. Notice that, if we compute the accuracy considering just the closest tower to a user’s actual home location as ground truth (i.e., $H^{(u)} = H_{1}^{(u)}$) the ranking of the most accurate combinations of streams and HDAs does not change significantly (Fig. 6). Again, XDRs lead to the highest average accuracy and HDAs based on weekdays and nighttime records are way the best ones, even though the average accuracy drops down.

The home detection accuracy of an HDA on a stream identifies home locations as the towers with the highest activity. We can relax this condition by defining a detected home location to be correct if at least one of the top *k* towers detected by an HDA *X* on a stream *T* is correct. We denote with $H^{(k)}_{X, T}(u)$ a tower that is within the *k* towers with the highest activity according to HDA *X* and stream *T* for user *u*. We define the *home detection k-accuracy* ($\mathrm{ACC} ^{(k)}_{X, T}$) of the combination of HDA *X* and stream *T* as the number of $H^{(k)}_{X, T}(u)$ over the total number of ground truth users $|G|$. Given this definition, $\mathrm{ACC} _{X, T} = \mathrm{ACC}^{(1)}_{X, T}$. Figure 6b-c shows $\mathrm{ACC} ^{(k)}_{X, T}$ for $k=2, 3$ and the top five HDAs of each stream. See Supplementary Fig. 2 for the *k*-accuracy of all combinations of HDAs and data streams. $\mathrm{ACC} ^{(k)}_{X, T}$ increases with *k* and XDRs lead to the highest average accuracy ($\overline{\mathrm{ACC}}^{(2)}_{X, \mathrm{ XDRs}} = 0.78$, $\overline{\mathrm{ACC}}^{(3)}_{X, \mathrm{ XDRs}} = 0.85$). It is worth noting how, as *k* increases, DD climbs the ranking of the top five most accurate HDAs for XDRs and CPRs, until it becomes the best HDA for 3-accuracy.

### Distance to actual home

**Figure 7:**
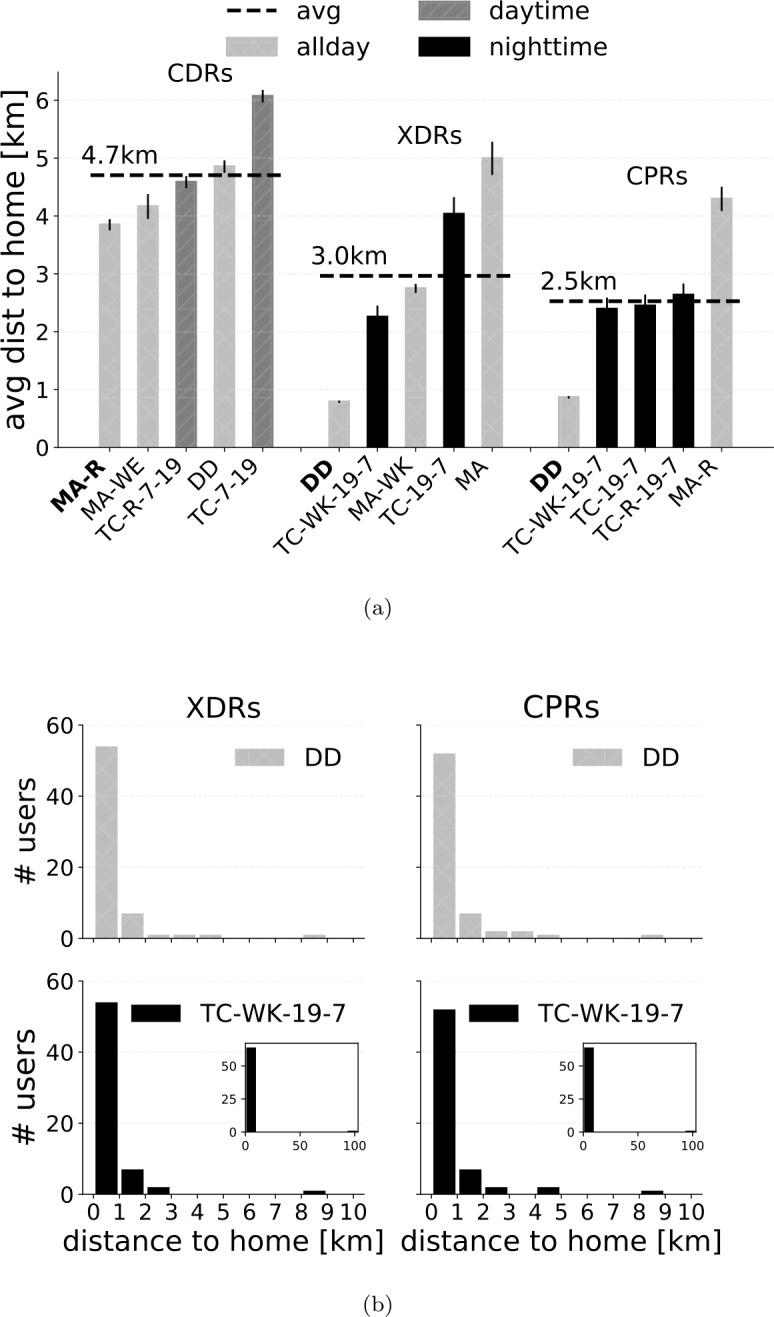
(**a**) Average distance between the home location detected by each HDA ($k=1$, any of the three closest towers) on each stream and the actual user’s home location. (**b**) Distribution of the distance to the actual home for DD and TC-WK-19-7 on XDRs and CPRs

**Figure 8:**
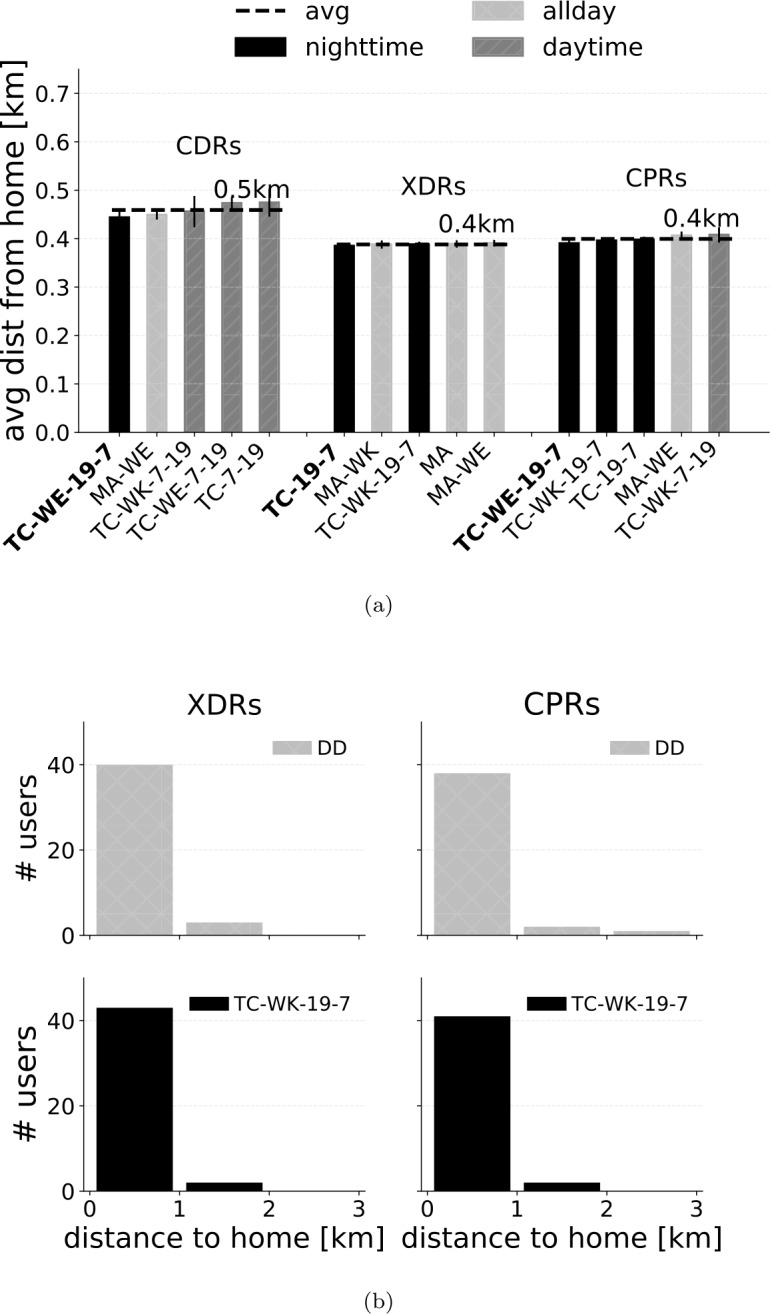
(**a**) Average distance between the home location detected by each HDA ($k=1$, any of the three closest towers) on each stream and the actual user’s home location considering only those users for which the home is correctly detected. (**b**) Distribution of the distances to the actual home for DD and TC-WK-19-7 on XDRs and CPRs, considering only those users for which the home is correctly detected

For each user, we compute the distance of the home tower detected by a given combination of stream and HDA to their actual home location. Figure 7a shows the five HDAs with lowest average distance for the three streams. The average distance of the top HDAs is in general lower than 5 km. Moreover, CPRs guarantee the lowest average distance overall. Note that DD is slightly better than TC-WK-19-7 in this regard, for both XDRs and CPRs. To investigate this aspect more deeply, we compute the distribution of the distance to home for DD and TC-WK-19-7 on XDRs and CPRs (Fig. 7b). We find that, for just one user, TC-WK-19-7 detects a home location that is 100 km away to the actual one, leading to an average distance higher than DD. However, if we compute the distribution of distance to home only for those users whose home location is correctly detected, TC-WK-19-7 achieves a lower average distance than DD, resulting in the best HDA (Fig. 8). We do not find any significant difference in the results by normalizing the distance to the actual home by the average distance among the three closest towers to it, a proxy for the density of towers in the area where the actual home is located (see Supplementary Information 3).

### Data minimization

We then turn to the question of what is the minimum amount of data, for each stream, that is required to achieve a given sought accuracy, and whether they are qualitatively different at the time of doing so. For instance, do CPRs work better with less records than XDRs, or vice versa? This experiment is important for both researchers and telco operators: it is usually not easy to access mobile phone data. Thus, requesting these data with a principled approach rather than a “blanket” request, could go a long way in building trust between companies and researchers, while also requiring less human resources and storage infrastructure to prepare and share the data. To learn more about this, we run two randomization algorithms.

**Figure 9:**
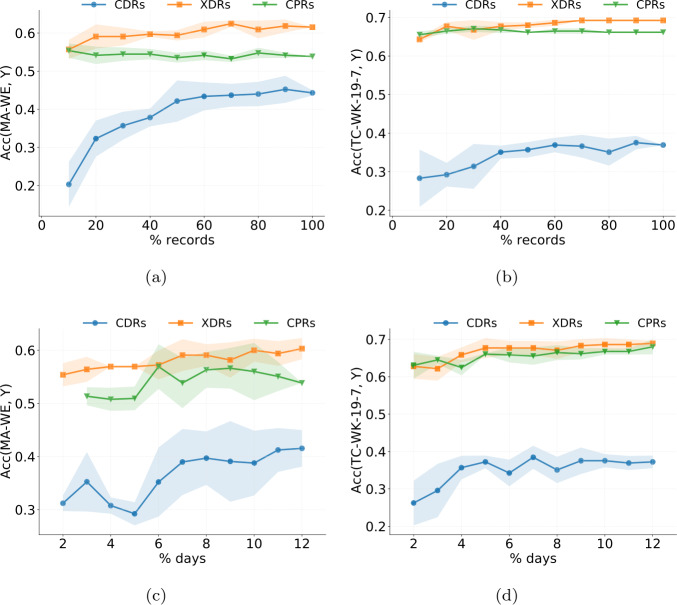
Results of the first data minimization experiment. (**a**), (**c**) Number of records randomly selected versus the average and standard deviation of home detection accuracy ($k=1$, any of the three closest towers) for TC-WK-19-7 and MA-WE. (**b**), (**d**) Number of days randomly selected versus the average and standard deviation of home detection accuracy ($k=1$, any of the three closest towers) for TC-WK-19-7 and MA-WE

The first one is based on sampling a random fraction of user records, for different values of the sampled fraction. We compute $\mathrm{ACC} _{X,T}$ for 10%, 20%, …, 100% of the available records for each user, and run this experiment five times to obtain multiple realizations of the sampling process. Figure 9a,c shows the average detection accuracy varying the number of records used for TC-WK-19-7 and MA-WE (the HDAs with the best accuracy on XDRs/CPRs and CDRs, respectively). The shaded areas indicate the standard deviation over the five realizations.

We find three main results. First, while for CDRs the accuracy largely varies based on the set of records selected per user, XDRs and CPRs guarantee more stable accuracy. Second, a high fraction of CDRs is needed to achieve an accuracy similar to that obtained using 100% of the records. For example, for MA-WE, at least 50% of the records are needed (Fig. 9a). In contrast, a smaller fraction of XDRs is sufficient to achieve the same accuracy as using all the records. Third, XDRs perform the best regardless of the fraction of records selected, highlighting *a fortiori* that they should be preferred over the other two streams when performing home location detection.

The second randomization algorithm samples whole days from each user’s timeline, and only considers records for the selected days. Thus, we calculate the home detection algorithms accuracy for all records of two days, three days, …, 12 days of the available ones, and run this experiment five times to obtain multiple realizations of the sampling process. Figure 9b,d shows the average home detection accuracy varying the number of days used for TC-WK-19-7 and MA-WE. The results confirm those of the first experiment: *(i)* CDRs lead to less stable accuracy than XDRs and CPRs; *(ii)* just a small fraction of days is enough for XDRs and CPRs to achieve an accuracy similar to using all available days; *(iii)* XDRs are the best stream regardless of the days that were selected. Supplementary Information 4 reports the results of both data minimization experiments for all the other HDAs.

## Conclusions

Mobile phone data are a crucial data source for official statistics, with most of the tasks involved including the identification of a device’s home location. In this paper, we provided an thorough evaluation of existing HDAs on an individual ground truth data set. Our experiments reveal that the type of stream used - CDRs, XDRs, CPRs - heavily influences the accuracy of the home detection. Similarly, the choice of the algorithm is crucial: algorithms based on weekdays and/or nighttime records – such as TC-WK-19-7 (records between 7 pm and 7 am during weekdays) and TC-19-7 (all records between 7 pm and 7 am) – are the most accurate algorithms, regardless of the stream. As a consequence, using XDRs in combination with TC-WK-19-7 is the solution leading the highest home detection accuracy and the lowest distance to the actual home overall. In contrast, HDAs based on daytime records or spatial perimeter should be avoided given their low accuracy.

Nevertheless, our data minimization experiment revealed that XDRs and CPRs are more resilient than CDRs to the reduction of the number of records available per user, which also impact the number of ties in towers identified as homes. Overall, our work demonstrates that CDRs, the most used stream in the literature to detect individuals’ home, at least nowadays, lead to low accuracy and stability, and should be handled more carefully.

As future work, we plan to exploit our ground truth dataset to investigate most sophisticated solutions to home location detection. For example, rather than just relying on the records related to a tower, one could exploit the entire spatio-temporal information contained in the full trajectory of a user. In this regard, deep learning approaches to human mobility [23] have the potential to uncover complex patterns and boost the accuracy of home location detection.

Our study has also several limitations. First, the sample size is relatively small at 65 participants. However, participants consented to giving us all their digital traces for two weeks, providing high coverage and fine resolution of individual behaviors. Still, in future research, it would be desirable to have a larger and more representative sample of participants consenting to these kind of study. Similarly, since for privacy reasons the participants did not provide us any demographic information (e.g., gender, age), the algorithms may be under/overestimating activity for poorer versus richer participants, or maybe some distinguishing behaviors given gender or age. We hope all these shortcomings will be addressed by the community or even ourselves in future work with better access to data. Finally, since all participants were employed and worked during business hours, our study is biased towards people with the associated mobility routine. We hope to come back to this problem in the near future.

In the meantime, experiences like ours may contribute to shape the discussion on what the best mobile phone stream is to capture presences and human mobility patterns. This is crucial because the decisions of citizens and policy makers depend on what we measure, how good our measurements are and how well our measures are understood with the ultimate goal of building a more consistent and comparable body of knowledge.

## Supplementary Information

Below is the link to the electronic supplementary material.

The pdf file entitled “Supplementary material for Evaluation of Home Detection Algorithms on Mobile Phone Datausing Individual-Level Ground Truth” contains extra details about the computation carried out in the main text. (PDF 1.1 MB)

## Data Availability

The code of the HDAs is available at https://github.com/leoferres/home_loc.
