# Supplementary material for: Evaluation of home detection algorithms on mobile phone data using individual-level ground truth
Source: EPJ Data Sci. 2021 Jun 2;10(1):29. doi: 10.1140/epjds/s13688-021-00284-9 (PMC8170634; doi:10.1140/epjds/s13688-021-00284-9)
Supplement: Supplementary file 1 — The pdf file entitled “Supplementary material for Evaluation of Home Detection Algorithms on Mobile Phone Datausing Individual-Level Ground Truth” contains extra details about the computation carried out in the main text. (PDF 1.1 MB) [file 13688_2021_284_MOESM1_ESM.pdf]

Supplementary Material for Evaluation of Home Detection Algorithms on Mobile Phone Data using Individual-Level Ground Truth

Luca Pappalardo, Leo Ferres, Unidersidad del Desarrolo and
Telef'onica Chile,Manuel Sacasa, Ciro Cattuto, Loreto Bravo

I. SUPPLEMENTARY FIGURES

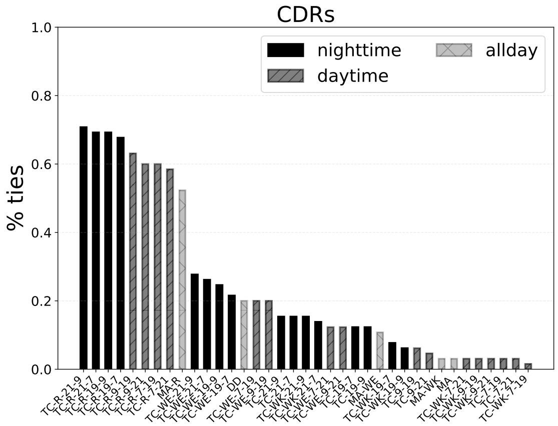

(a)

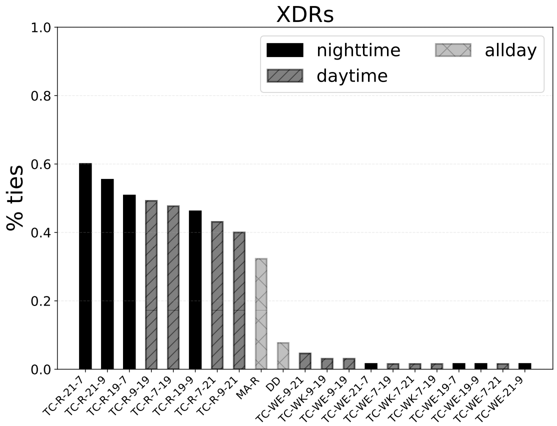

(b)

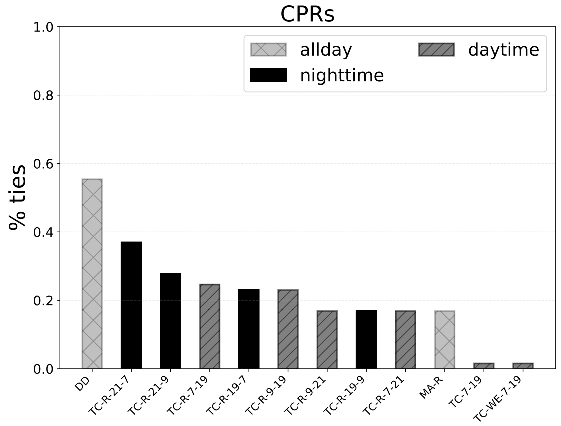

(c)

Supplementary Figure 1: Percentage of ties (number of users for which the most active towers have the same activity) for each HDA and data stream.

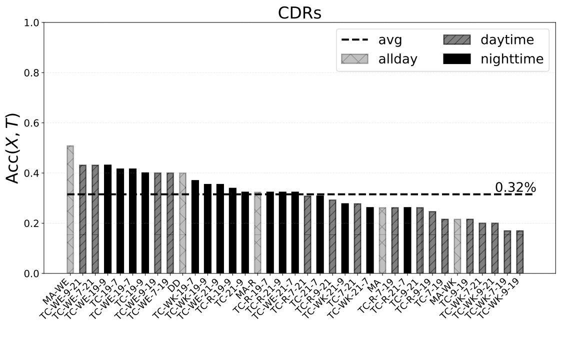

(a)

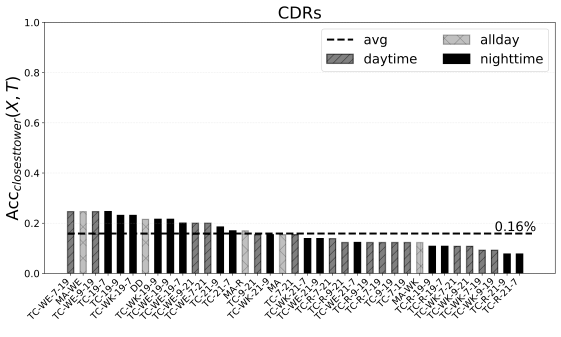

(b)

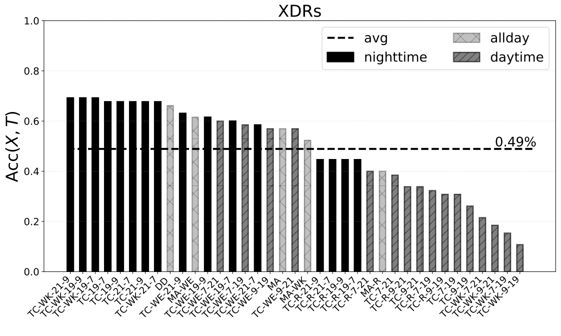

(c)

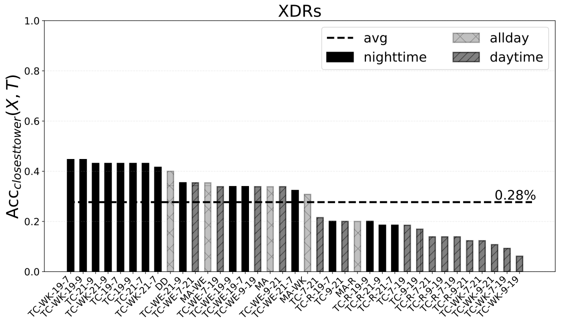

(d)

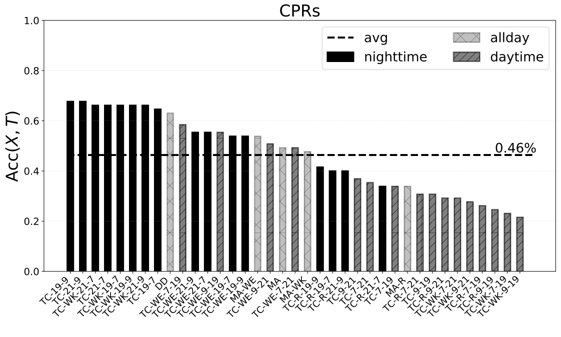

(e)

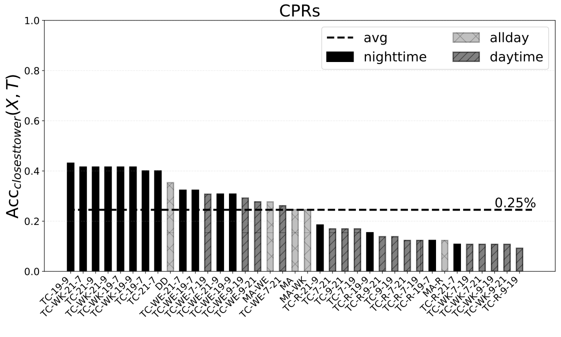

(f)

Supplementary Figure 2: (a, c, e) Home detection accuracy of each HDAs for each data stream. (b, d, f) Home detection accuracy of each HDAs considering just the nearest tower to the user's actual home as ground truth, for each data stream.

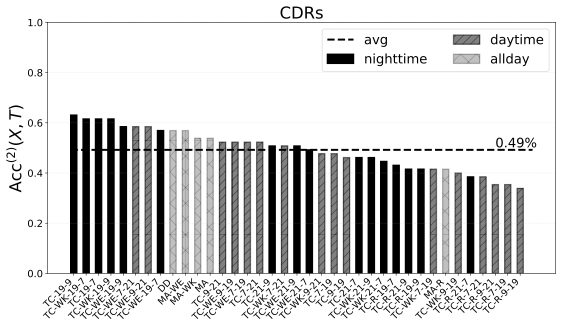

(a)

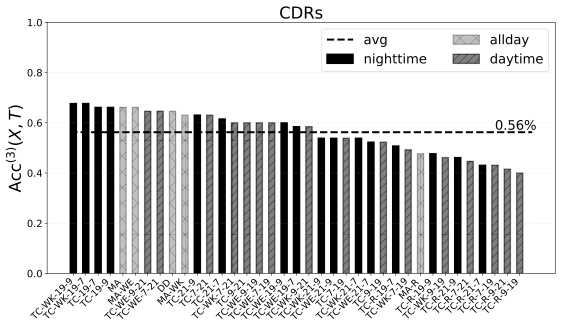

(b)

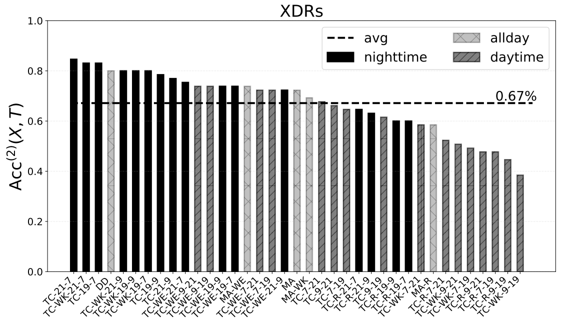

(c)

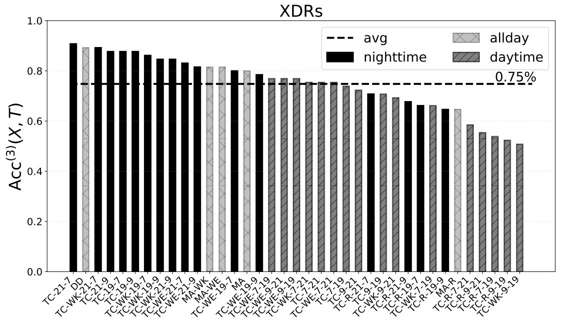

(d)

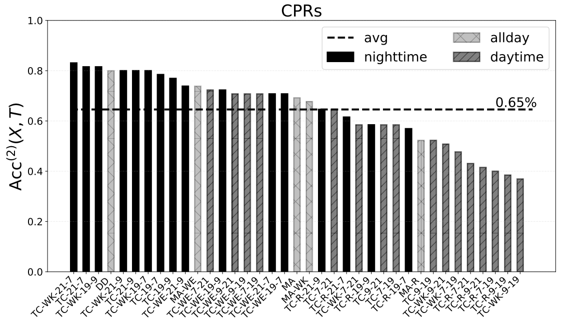

(e)

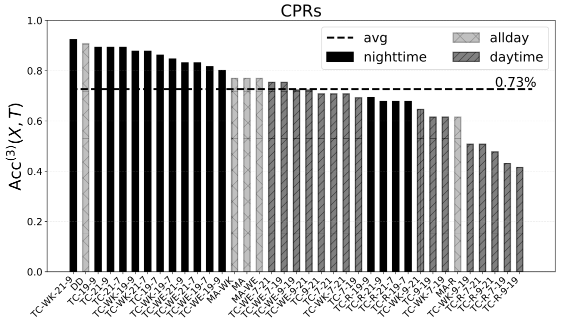

(f)

Supplementary Figure 3: (a, c, e) Home detection 2-accuracy of each HDAs for each data stream. (b, d, f) Home detection 3-accuracy of each HDAs for each data stream.

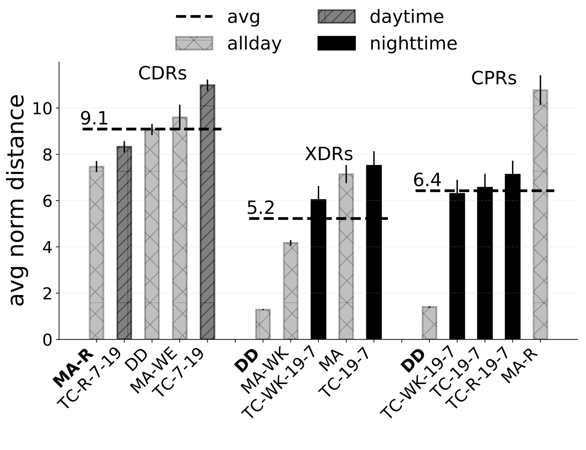

(a)

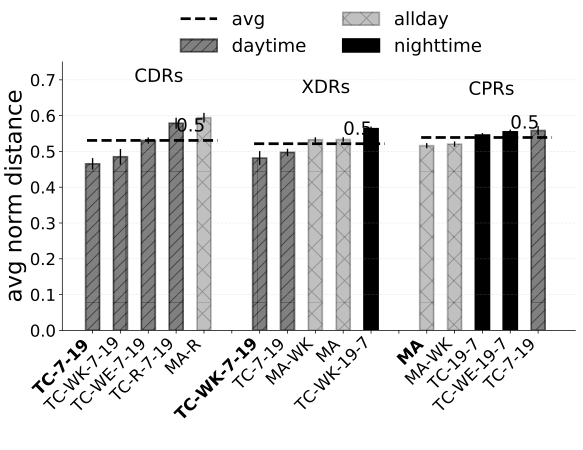

(b)

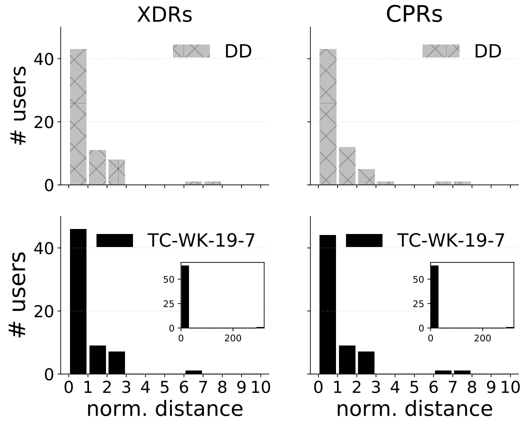

(c)

Supplementary Figure 4: (a) Average normalized distance between the home location detected by the top five HDAs and the actual home. (b) Average normalized distance between the home location detected by the top five HDAs and the actual home, considering only those users for which the home is correctly detected. (c) Distribution of normalized distances to the actual home for DD and TC-WK-19-7 on XDRs and CPRs.

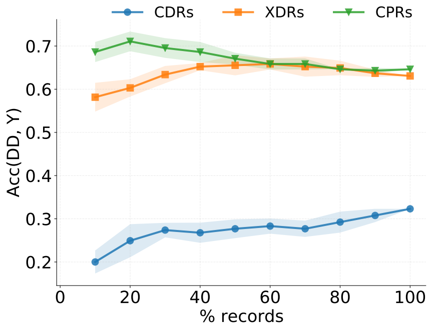

(a)

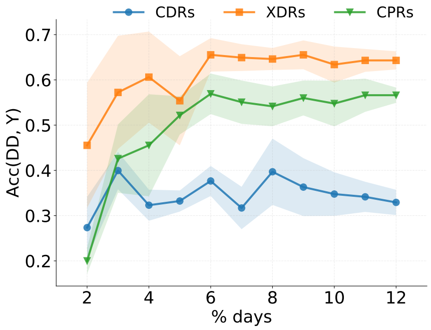

(b)

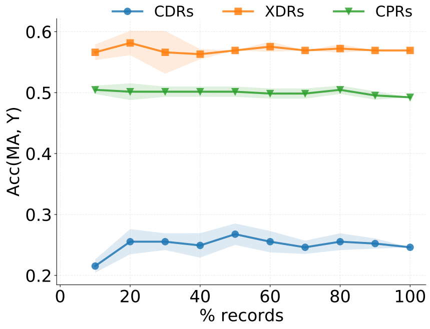

(c)

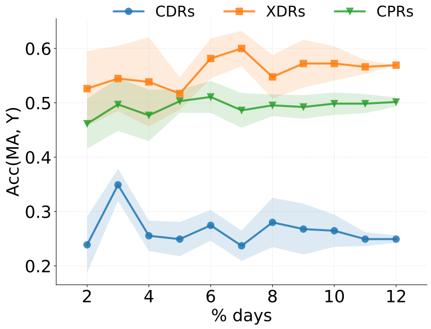

(d)

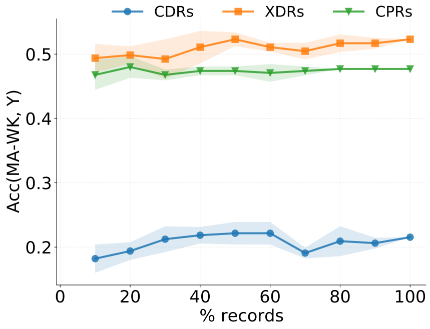

(e)

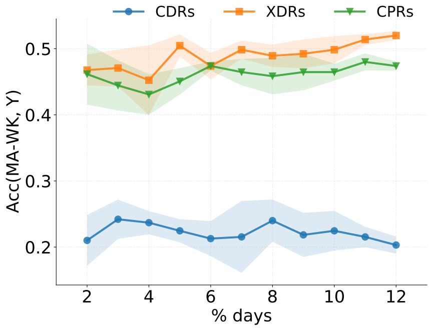

(f)

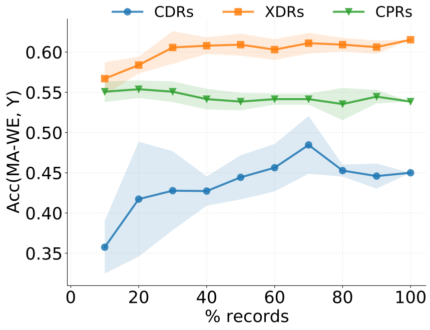

(g)

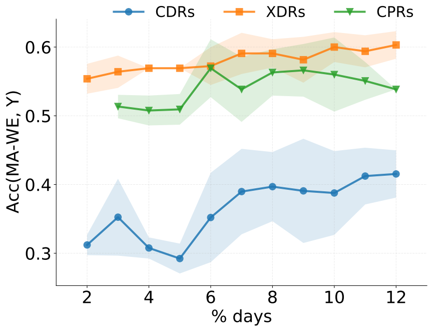

(h)

Supplementary Figure 5: Results of the data minimization experiments for MA-based HDAs and DD.

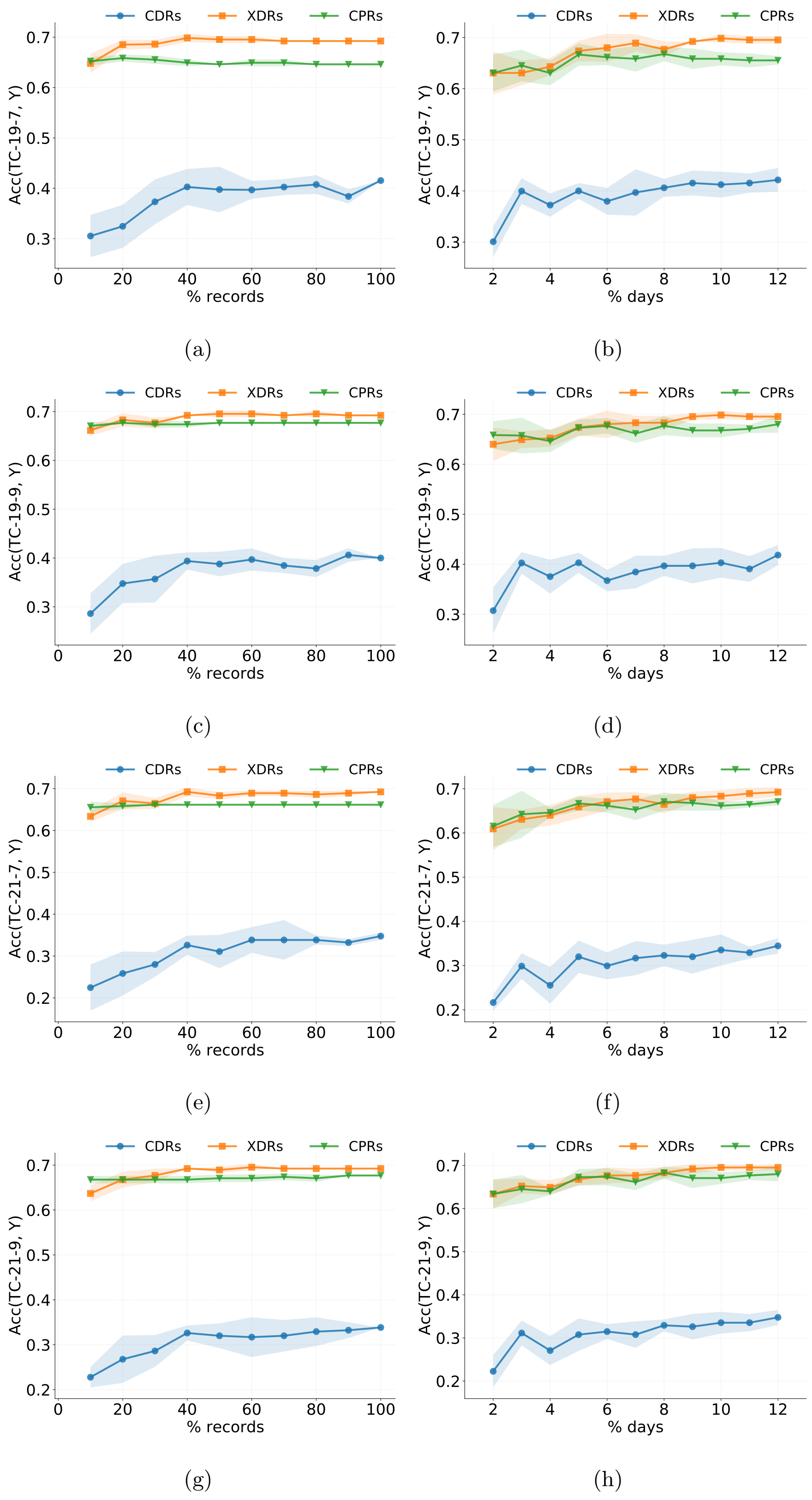

Supplementary Figure 6: Results of the data minimization experiments for HDAs based on records during nighttime hours.

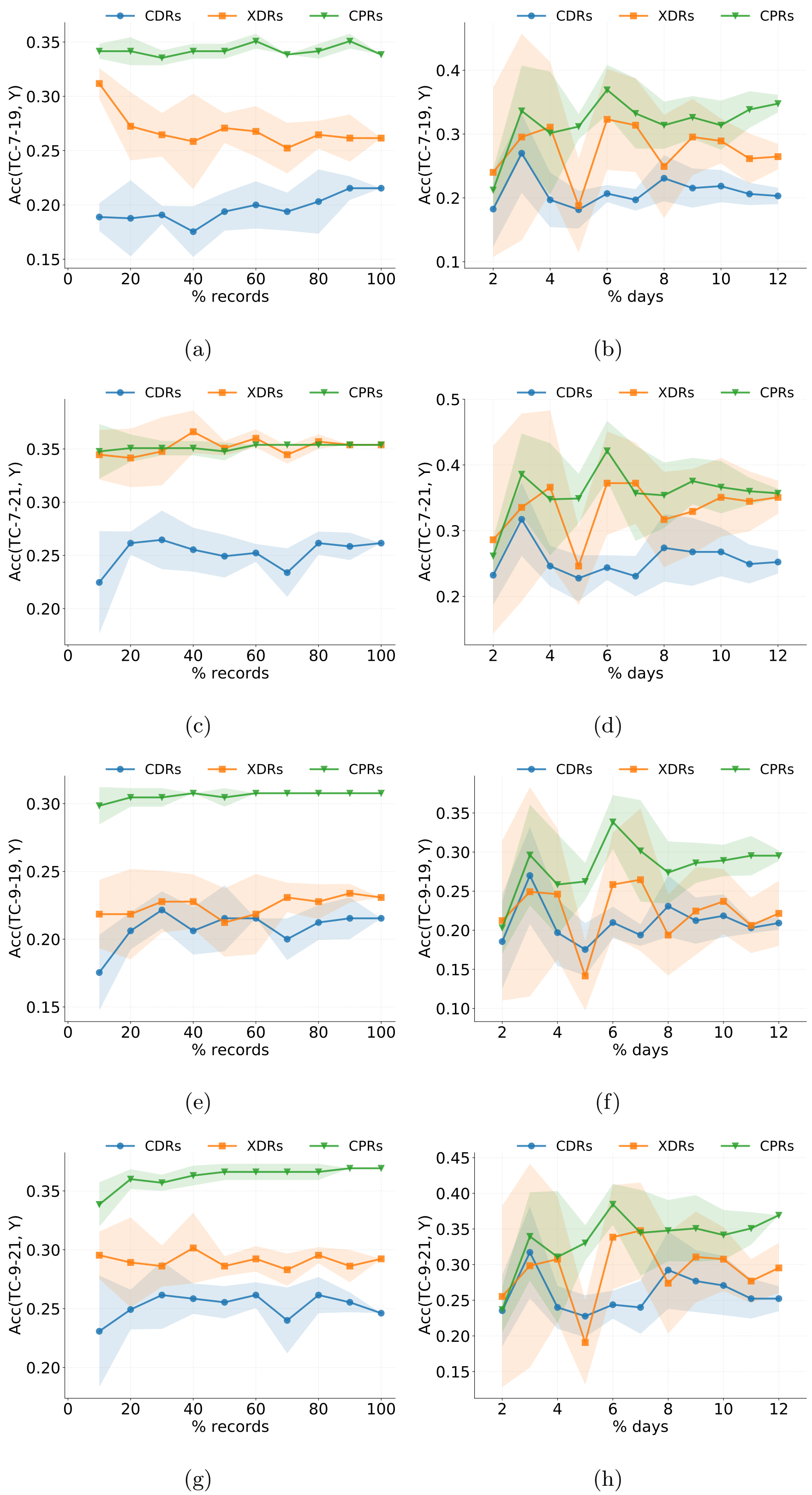

Supplementary Figure 7: Results of the data minimization experiments for HDAs based on records during daytime hours.

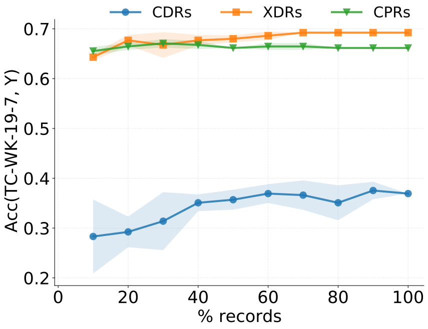

(a)

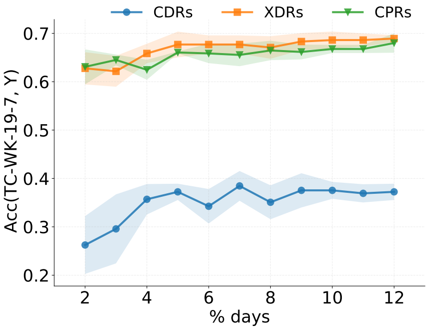

(b)

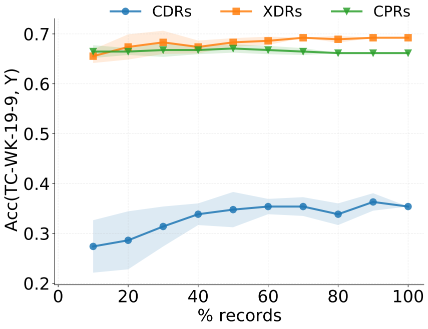

(c)

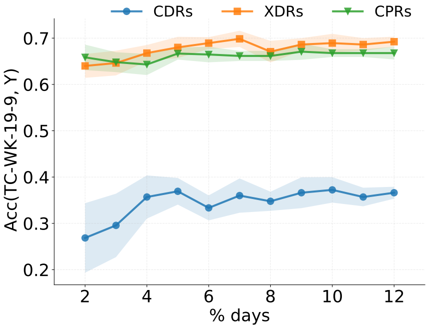

(d)

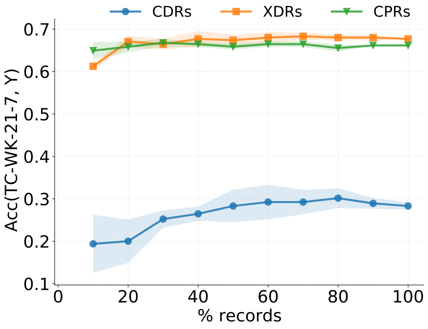

(e)

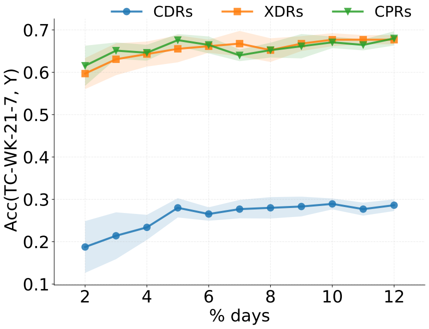

(f)

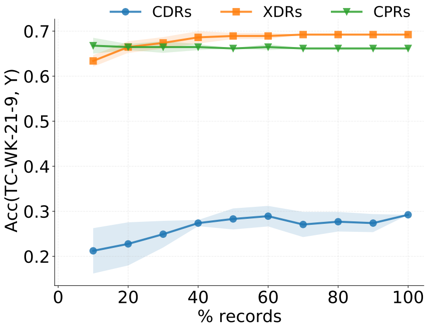

(g)

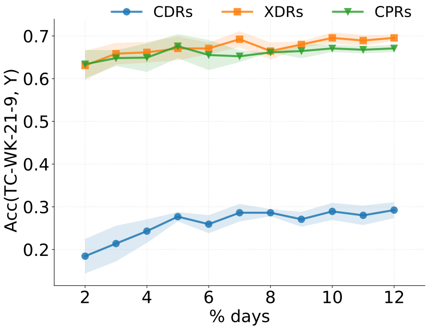

(h)

Supplementary Figure 8: Results of the data minimization experiments for HDAs based on records during weekdays and nighttime hours.

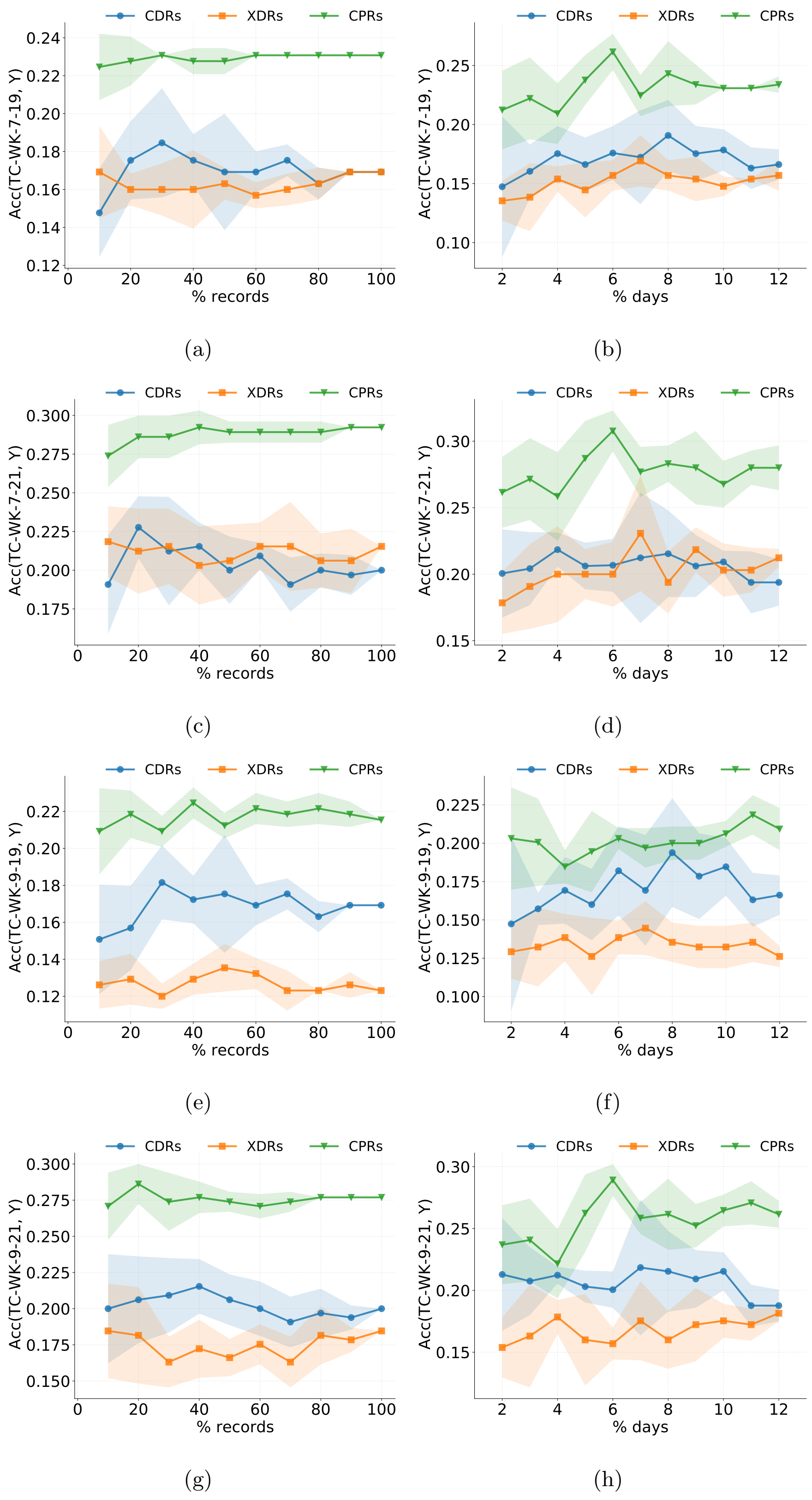

Supplementary Figure 9: Results of the data minimization experiments for HDAs based on records during weekdays and daytime records.

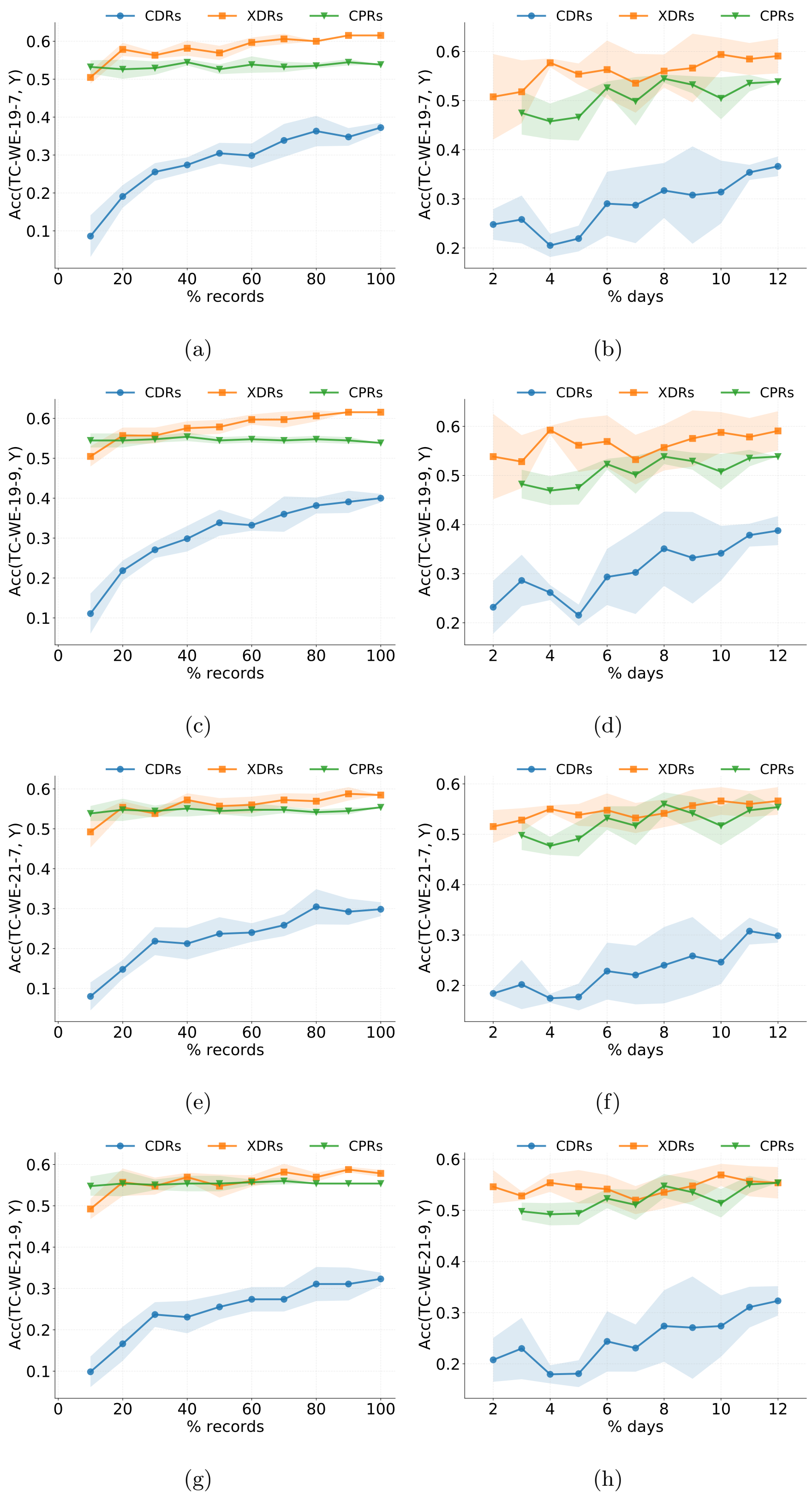

Supplementary Figure 10: Results of the data minimization experiments for HDAs based on records during weekend days and nighttime hours.

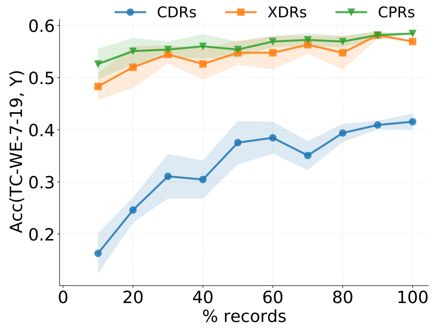

(a)

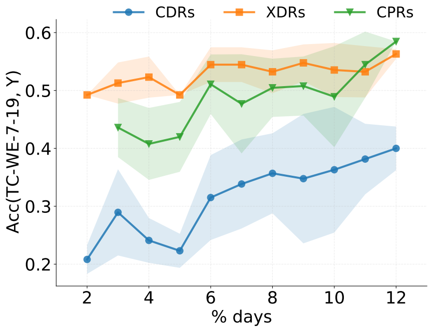

(b)

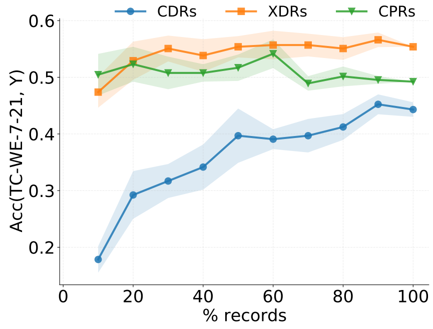

(c)

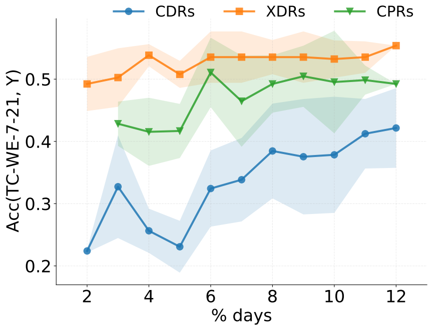

(d)

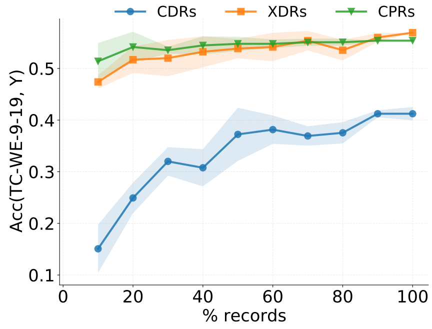

(e)

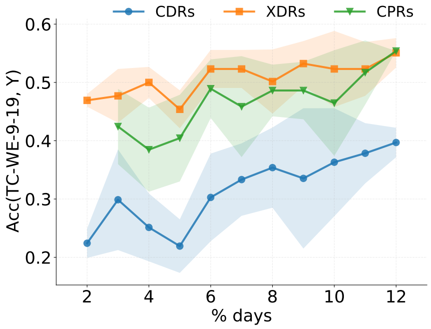

(f)

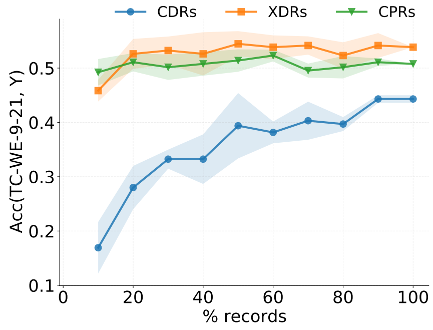

(g)

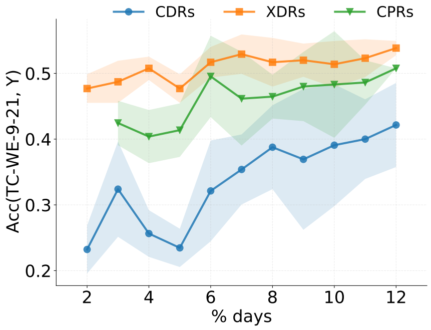

(h)

Supplementary Figure 11: Results of the data minimization experiments for HDAs based on records during weekend days and daytime hours.

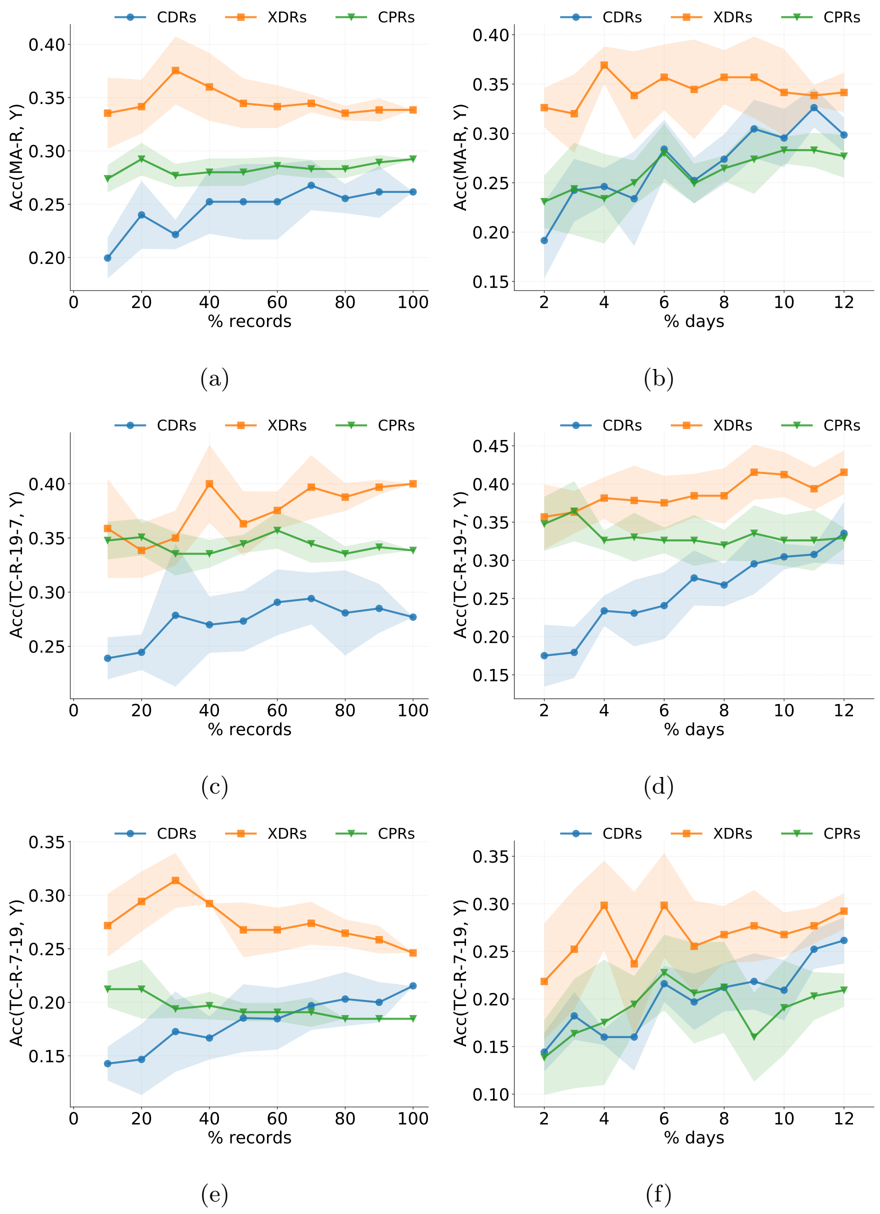

Supplementary Figure 12: Results of the data minimization experiments for HDAs that implement a perimeter of radius 1km around the tower.

II. SUPPLEMENTARY TABLE

	three nearest towers			nearest tower		
	CDRs	XDRs	CPRs	CDRs	XDRs	CPRs
DD	0.40	0.66	0.63	0.22	0.40	0.35
MA	0.26	0.57	0.49	0.15	0.34	0.25
MA-R	0.32	0.40	0.34	0.17	0.20	0.12
MA-WE	0.51	0.62	0.54	0.25	0.35	0.28
MA-WK	0.22	0.52	0.48	0.12	0.31	0.25
TC-19-7	0.42	0.68	0.65	0.25	0.43	0.40
TC-19-9	0.40	0.68	0.68	0.23	0.43	0.43
TC-21-7	0.31	0.68	0.66	0.17	0.43	0.40
TC-21-9	0.32	0.68	0.68	0.18	0.43	0.42
TC-7-19	0.22	0.31	0.34	0.12	0.18	0.17
TC-7-21	0.28	0.38	0.35	0.15	0.22	0.17
TC-9-19	0.22	0.26	0.31	0.12	0.17	0.14
TC-9-21	0.26	0.34	0.37	0.15	0.20	0.17
TC-R-19-7	0.32	0.45	0.40	0.11	0.20	0.12
TC-R-19-9	0.34	0.45	0.42	0.11	0.20	0.15
TC-R-21-7	0.26	0.45	0.34	0.08	0.18	0.11
TC-R-21-9	0.32	0.45	0.40	0.08	0.18	0.18
TC-R-7-19	0.26	0.32	0.26	0.12	0.14	0.12
TC-R-7-21	0.31	0.40	0.31	0.14	0.14	0.12
TC-R-9-19	0.25	0.31	0.25	0.12	0.14	0.09
TC-R-9-21	0.29	0.34	0.29	0.12	0.12	0.14
TC-WE-19-7	0.42	0.60	0.54	0.20	0.34	0.32
TC-WE-19-9	0.43	0.62	0.54	0.22	0.34	0.31
TC-WE-21-7	0.32	0.58	0.55	0.12	0.32	0.32
TC-WE-21-9	0.35	0.63	0.55	0.14	0.35	0.31
TC-WE-7-19	0.40	0.58	0.58	0.25	0.34	0.31
TC-WE-7-21	0.43	0.60	0.49	0.20	0.35	0.26
TC-WE-9-19	0.40	0.57	0.55	0.25	0.34	0.29
TC-WE-9-21	0.43	0.57	0.51	0.20	0.34	0.28
TC-WK-19-7	0.37	0.69	0.66	0.23	0.45	0.42
TC-WK-19-9	0.35	0.69	0.66	0.22	0.45	0.42
TC-WK-21-7	0.26	0.68	0.66	0.14	0.42	0.42
TC-WK-21-9	0.28	0.69	0.66	0.15	0.43	0.42
TC-WK-7-19	0.17	0.15	0.23	0.09	0.09	0.11
TC-WK-7-21	0.20	0.22	0.29	0.11	0.12	0.11
TC-WK-9-19	0.17	0.11	0.22	0.09	0.06	0.11
TC-WK-9-21	0.20	0.18	0.28	0.11	0.11	0.11

TABLE I: Accuracy of all 37 HDAs considered in our study.

III. SUPPLEMENTARY INFORMATION

Supplementary Information 1: Activity ties

We investigate the percentage of ties generated by an HDA, i.e., fraction of users for which several towers have the same highest activity. Supplementary Figure 1 shows the percentage of ties that may occur for each HDA and stream. We observe two main results. First, CDRs are the worst stream in this regard, as they lead to the highest percentage of ties overall and the highest number of HDAs with at least one user with a tie. In contrast, CPRs and XDRs not only lead to a lower average percentage of ties, but also to a lower number of HDAs with at least one tie. Second interesting result is that the HDAs with a significant percentage of ties are those involving the 1km radius (percentage of ties higher than 25%, see Supplementary Figure 1).

Supplementary Information 2: Accuracy of all HDAs

Supplementary Table I (columns under "three nearest towers") and Supplementary Figure 2a,c,e show the accuracy of all 37 HDAs. For CDRs, MA-WE is the best HDA. For XDRs the best algorithms are (on par) TC-WK-21-9, TC-WK-19-9, and TC-WK-19-7 (time constraints based on weekdays records and nighttime hours). For CPRs, the best HDAs are (on par) TC-19-9 and TC-21-9 (time constraints based on nighttime hours).

Supplementary Table I (columns under "nearest tower") and Supplementary Figure 2b,d,f show the accuracy of HDAs if just the closest tower to a user's actual home location is considered. Results are similar to those on left side of the Figure: for CDRs, the best HDAs are TC-WE-7-19, MA-WE, TC-WE-9-19, and TC-19-7 (on par); for XDRs, the best HDAs as TC-WK-19-7 and TC-WK-19-9 (on par); for CPRs, the best HDA is TC-19-9.

Supplementary Figure 3 shows the 2-accuracy and the 3-accuracy for all HDAs. The results confirm what we observe for the accuracy in Supplementary Figure 2.

Supplementary Information 3: Normalized distance to actual home

Figure 4a,b shows the average normalized distance of the home location detected by an HDA to the user's actual home location. The normalized is performed by dividing the distance between the detected location and the actual home location by the average distance among the three closest towers to the user's actual home location. Figure 4c shows the distribution of the normalized

distance between the detected home tower and the user's actual home for DD and TC-WK-19-7 on XDRs and CPRs.

Supplementary Information 4: Data minimization experiments

Figures 5-12 show the results of the minimization experiments for all HDAs and streams.
